# Characterization and Expression Analysis of Regeneration-Associated Protein (Aj-Orpin) during Intestinal Regeneration in the Sea Cucumber *Apostichopus japonicus*

**DOI:** 10.3390/md20090568

**Published:** 2022-09-06

**Authors:** Fang Su, Lina Sun, Xiaoni Li, Wei Cui, Hongsheng Yang

**Affiliations:** 1 CAS Key Laboratory of Marine Ecology and Environmental Sciences, Institute of Oceanology, Chinese Academy of Sciences, Qingdao 266071, China; 2 Laboratory for Marine Ecology and Environmental Science, Qingdao National Laboratory for Marine Science and Technology, Qingdao 266237, China; 3 Center for Ocean Mega-Science, Chinese Academy of Sciences, Qingdao 266071, China; 4 CAS Engineering Laboratory for Marine Ranching, Institute of Oceanology, Chinese Academy of Sciences, Qingdao 266071, China; 5 University of Chinese Academy of Sciences, Beijing 100049, China; 6 Shandong Province Key Laboratory of Experimental Marine Biology, Qingdao 266071, China; 7 The Innovation of Seed Design, Chinese Academy of Sciences, Wuhan 430071, China

**Keywords:** sea cucumber (*Apostichopus japonicus*), regeneration, holothurians, gene function, Aj-Orpin

## Abstract

*Apostichopus japonicus* achieves intestinal regeneration in a short period after evisceration, and multiple genes are involved in this process. The transcriptome of *A. japonicus* was screened for regeneration-associated protein (Aj-Orpin), a gene that is specifically upregulated during intestinal regeneration. The expression and function of Aj-Orpin were identified and investigated in this study. The 5′ and 3′ RACE polymerase chain reaction (PCR) was used to clone the full-length cDNA of Aj-Orpin. The open reading frame codes for a 164 amino-acid protein with an EF-hand_7 domain and overlapping signal peptides and transmembrane regions. Moreover, Aj-Orpin mRNA and protein expression during intestinal regeneration was investigated using real-time quantitative PCR and Western blot. The expression pattern of Aj-Orpin in the regenerating intestine was investigated using immunohistochemistry. The results showed that Aj-Orpin is an exocrine protein with two EF-hand-like calcium-binding domains. Expression levels were higher in the regenerating intestine than in the normal intestine, but protein expression changes lagged behind mRNA expression changes. Aj-Orpin was found to play a role in the formation of blastema and lumen. It was primarily expressed in the serosal layer and submucosa, suggesting that it might be involved in proliferation. These observations lay the foundation for understanding the role of Orpin-like in echinoderm intestinal regeneration.

## 1. Introduction

*Apostichopus japonicus* belongs to deuterostome, Echinodermata, a phylum at the branch between chordate and achordate, which indicates that it has special status in evolution. In response to environmental stress, it can eviscerate the intestine, respiratory tree, gonad, and all internal organs, followed by the recovery of functional internal organs in about three weeks [1]. Due to its excellent regeneration ability, it is considered as another model for studying regeneration [2]. The histology and cytology of sea cucumber regeneration have been extensively studied [3,4]. The blind end heals and forms the blastema after evisceration, and the lumen opens along the thickened mesentery’s end. In about two weeks, a connected fragile intestine can be formed. Researchers agreed that intestinal regeneration in holothurians could be divided into five stages based on morphological and histological changes: wound healing (0–3 days post-evisceration, dpe), blastema formation (3–7 dpe), lumen formation (7–14 dpe), intestinal differentiation (14–21 dpe), and intestinal growth (21 dpe) [5,6,7]. After evisceration, differentiated cells in the mesothelial and connective tissue layers dedifferentiate and transdifferentiate, then migrate to the free end of the mesentery to begin regeneration [8,9,10,11,12]. Mesothelial migration is accompanied by collagen degradation and matrix metalloproteinase activation in connective tissue, a process known as extracellular matrix (ECM) remodeling [9,13,14,15]. Proliferation is similar to the pattern of dedifferentiation and ECM remodeling, and it is distributed in a gradient along the mesentery with higher levels at the distal end [5,9,13].

Microvesicles and secretory vacuoles indicate that dedifferentiated cells have completed their redifferentiation and are forming intestinal luminal epithelium [11,16]. This is the process of intestinal regeneration, which is first dominated by morphallaxis and then epimorphosis [4,17].

To investigate the mechanism of regeneration at higher throughput, researchers have constructed EST libraries [18,19], gene expression profiles [20,21], and regeneration transcriptomes [22,23]. The genome’s construction lays the foundation for dissecting the molecular mechanisms of intestinal regeneration [24]. MicroRNAs are involved in specific cellular events (cell proliferation, migration, and apoptosis), metabolic regulation, and energy redistribution, according to noncoding RNA analysis [25]. In addition to omics research, key signaling pathways are being investigated, such as the Wnt signaling pathway, which is the only one under positive selection in regenerating echinoderms [26,27]. Wnt pathway is involved in the activation of gene regulatory networks during development and regeneration [6]. They are transduced to the canonical pathway for cell fate determination, and to the noncanonical pathway for control of cell movement and tissue polarity. Although regeneration is a multi-gene regulated process, only a few genes, such as Frizzled, WntA, Wnt9, Bmp1, Ependymin, serum amyloid A, survivin, mortalin, and matrix metalloproteinase genes, have been studied in depth [14,28,29,30,31,32].

According to transcriptome and expression profile studies, the regeneration-associated protein (Aj-Orpin) was involved in the intestinal regeneration of *A. japonicas* [23]. The mRNA expression level of Aj-Orpin was measured at 3, 7, 14, and 21 days post-evisceration, significantly upregulated up to 200–400 times compared to the normal state, and then gradually decreased to the normal state at 14 and 21 days post-evisceration. In *Holothuria glaberrima*, the homologous gene Orpin showed no significant differential expression pattern in regenerating mesentery [33]. According to García-Arrarás, Orpin proteins are a group of secreted CaBPs with two EF-hands.

However, the level of Aj-Orpin protein expression in intestinal regeneration has not been reported, nor has the level of mRNA expression in the early stages of regeneration. There have been no further studies on Orpin-like genes’ functions, so that Aj-Orpin could be the first regenerative-specific gene to characterize function. We cloned the full-length of Aj-Orpin gene from *A. japonicus* and analyzed sequence characteristics to predict gene function in this study. Furthermore, we evaluated the levels of mRNA and protein expression, as well as the distribution of protein, at different stages of regeneration. Our findings lay the foundation for further research into the role of Orpin-like in echinoderm regeneration.

## 2. Results

### 2.1. Cloning and Sequence Analysis of Aj-Orpin Full-Length cDNA in A. japonicus

**Figure 1:**
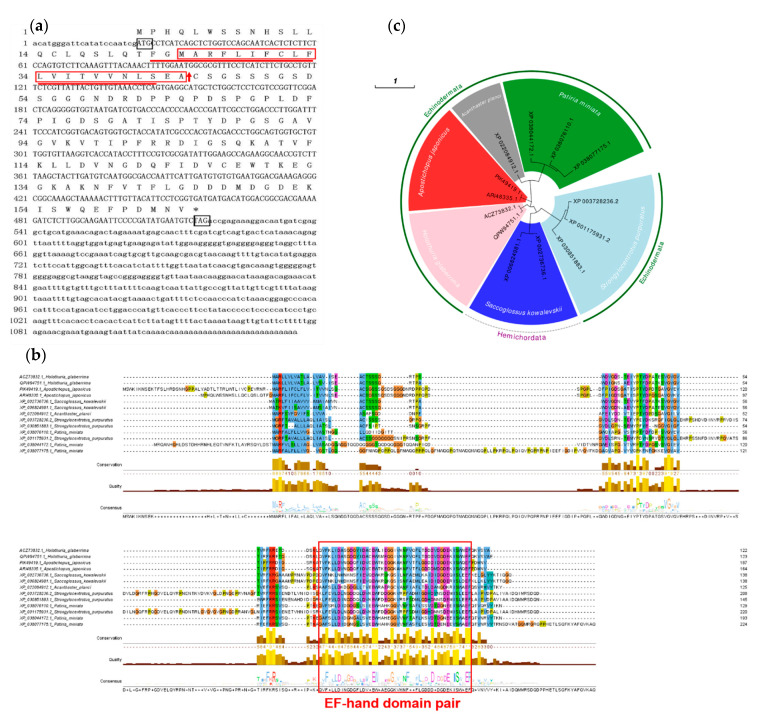
Sequence analysis of Aj-Orpin in *A. japonicus*. (**a**) Nucleotide sequence (1136 bp) of the Aj-Orpin gene (accession No. ARI48335.1) aligned with the predicted amino acid (AA) sequence (capital single-letter code). The full-length nucleotide sequence includes 22 bp 5′-untranslated region (UTR), a 619 bp 3′-UTR, and a 495 bp open-reading frame (164 AA). The start and stop codons are included in the black box. The stop codon is indicated by an asterisk. The signal peptide and the transmembrane region are denoted by the red box and the red underline respectively, and the cleavage site is shown with the red arrow. (**b**) Multiple alignment of the deduced amino acid sequences of Aj-Orpin. The EF-hand domain pair is denoted by the red box. (**c**) Phylogenetic trees based on Aj-Orpin amino acid sequences from *A. japonicus* and other species. The tree was constructed based on the multiple sequences generated by MUSCLE algorithm and aligned using the Maximum Likelihood method with 100 bootstraps.

Aj-Orpin’s full-length cDNA sequence (GenBank accession number: ARI48335.1) was 1136 bp long and contained a 22 bp 5′-untranslated region (UTR), a 619 bp 3′-UTR, and a 495 bp ORF encoding a 164 amino-acid protein with a predicted MW of 17.68 kDa and a theoretical IP of 4.31. EF-hand_7 is a domain found in the protein sequence. According to García-Arrarás’s report [33], the Aj-Orpin protein was predicted to have a signal peptide of 21 amino acids starting from the second methionine at the N-terminal (Figure 1a, as the red box shows). SEA-CS was the cleavage site between positions 21 and 22 (Figure 1a shows the red arrow). The transmembrane region of the sequence was between 22–42 amino acids predicted by Expasy (Figure 1a as the red underline showing).

BLAST analysis revealed that Aj-Orpin homologous sequences were only found in echinoderms and *Saccoglossus kowalevskii* (Hemichordata: Enteropneusta: Harrimaniidae). In *A. japonicus*, *Holothuria glaberrima* (QPW94751.1, identity 58.7%, coverage 55%; ACZ73832.1, identity 56.52%, coverage 55%), and *S. kowalevskii* (XP_006824981.1, identity 40.22%, coverage 51%; XP_002736736.1, identity 36.08%, coverage 54%), Orpin genes were double-copied. *A. japonicus* PIK49419.1 shared 94.33% identity and 85% coverage with Aj-Orpin. *Strongylocentrotus purpuratus* (XP_001175931.2, identity 38.55%, coverage 46%; XP_003728236.2, identity 38.46%, coverage 43%; XP_030851883.1, identity 37.5%, coverage 58%) and *Patiria miniata* (XP_038044172.1, identity 44.19%, coverage 51%; XP_038076110.1, identity 42.68%, coverage 49%; XP_038077175.1, identity 34.48%, coverage 61%) both had three copies. Only one gene was discovered in *Acanthaster planci* (XP_022084912.1, identity 49.45%, coverage 54%). The above amino acid sequences were subjected to multiple sequence alignments and a CDD conserved domain search. The findings revealed that they all had an EF-hand_7 domain from the EF-hand domain pair, implying that they may have similar functions (Figure 1b). According to the phylogenetic tree analysis of the above 13 sequences, the sequence homology of the same species was the highest, and Aj-Orpin clustered with homologs in *H. glaberrima* with high bootstrap probability (Figure 1c).

### 2.2. Aj-Orpin Gene Expression Pattern at the mRNA and Protein Level during Intestine Regeneration

**Figure 2:**
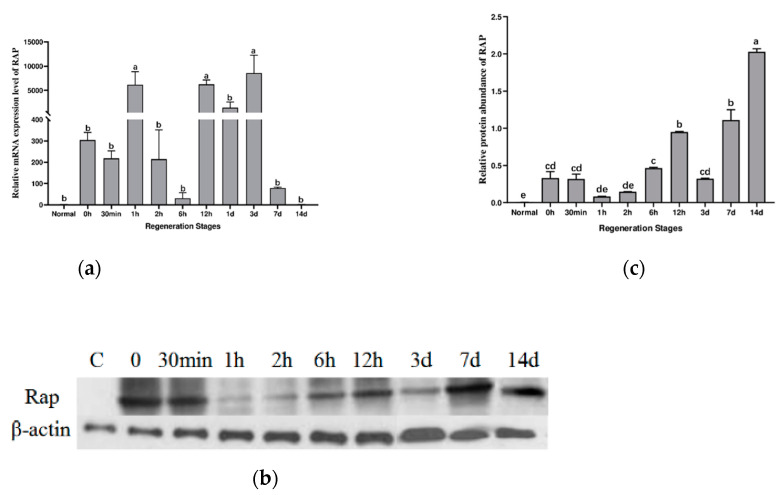
Relative expression levels of Aj-Orpin mRNA and protein during intestinal regeneration. (**a**) Real-time quantitative PCR analysis of Aj-Orpin expression at different intestinal regeneration time points: control, 0, 30 min, 1, 2, 6, 12 h and 1, 3, 7, and 14 days post-evisceration. Normal intestine was treated as the control group. (**b**) Western blot analysis showed Aj-Orpin protein expression at different time points during intestinal regeneration. β-actin served as the reference protein. (**c**) The protein expression level was evaluated by calculating the gray value of the bands. Data are shown as the mean ± SE. The lowercase letters indicate statistically significant differences (*p* < 0.05).

The expression level of Aj-Orpin mRNA during intestine regeneration was investigated using qRT-PCR with NADH dehydrogenase as the reference gene (Figure 2a). The melting curve had only one peak, demonstrating the specificity of the amplified PCR products. The level of mRNA expression increased rapidly after evisceration and peaked at 1 h. Then it decreased, reaching a low point at 6 h, followed by rapidly increasing about 5000 times. The mRNA expression level of the Aj-Orpin gene was 100 times that of normal individuals at 7 dpe, and it has mostly returned to normal after 14 days.

According to Western blot results, the expression levels of Aj-Orpin protein in the newborn intestine were upregulated compared to the intact intestine. In contrast, the expression level of β-actin was essentially stable (Figure 2b,c). RAP protein expression was upregulated by more than 70 times immediately after evisceration and by more than 400 times at 14 dpe. The protein expressions increased from day 3 to day 14, indicating that Aj-Orpin protein was involved in forming blastema and lumen. Protein expression changes take longer than mRNA expression changes.

### 2.3. Spatiotemporal Pattern of Aj-Orpin Expression

**Figure 3:**
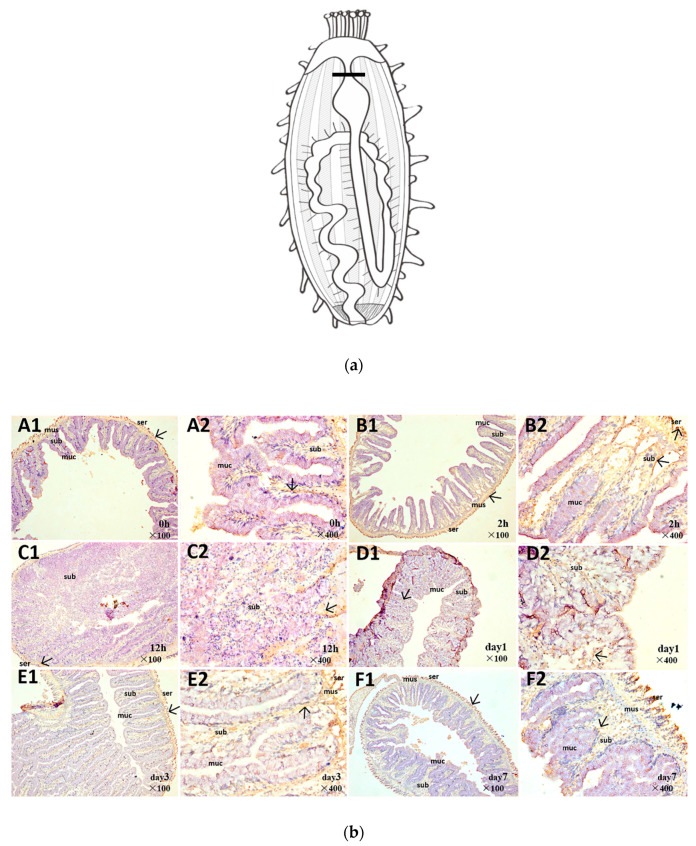
(**a**) Diagram to show the location of the slices. (**b**) Immunohistochemical staining for Aj-Orpin at different regeneration time points. (**A1**–**F1**) showed the pattern of Aj-Orpin protein expression from time 0 to day 7; (**A2**–**F2**) are the 4-fold magnification images of (**A1**–**F1**). ser: serosal layer, mus: muscle layer, sub: submucosal layer, muc: mucosal layer, lum: lumen. The reddish-brown particles pointed to by the arrows indicate positive Aj-Orpin expression. Aj-Orpin was mainly expressed in the serosal layer and submucosal layer and weakly in other layers.

The spatial expression of Aj-Orpin protein was demonstrated using immunohistochemistry at each stage of intestinal regeneration (A–F: 0, 2 h, 12 h, day1, day 3, and day 7). Specific positions (Figure 3a) were selected for crosscutting to describe the expression pattern of Aj-Orpin protein. Figure 3b depicts the outcomes (reddish-brown stain was a hybridization signal). The mucosal layer, the submucosal layer, the muscle layer, and the serosa layer are the four layers that make up the normal intestinal wall from inside out. Aj-Orpin protein was mostly expressed in the serosa, submucosal, and muscle layers during regeneration. The mucosal layer had very little signal. Aj-Orpin protein was strongly expressed in the serosa layer after evisceration. The signals in the submucosal layer became progressively weaker. From day 3 to day 7, the signals in the serosa layer became stronger. These results suggested that Aj-Orpin is involved in *A. japonicus* intestine regeneration.

## 3. Discussion

### 3.1. Aj-Orpin Is a Novel Secreted Calcium-Binding Protein (CaBP) Isolated from A. japonicus

For the first time, this study cloned and identified the full-length cDNA of Aj-Orpin from the sea cucumber *A. japonicus*. Sea cucumber (*Apostichopus japonicus*, *Holothuria glaberrima*), starfish (*Acanthaster planci*, *Patiria miniata*), sea urchin (*Strongylocentrotus purpuratus*), and acorn worm (*Saccoglossus kowalevskii*) all had homologous sequences, indicating that the gene were found in echinoderms and hemichordates. A previous study discovered similar sequences in *S. kowalevskii* and sea cucumber *H. glaberrima* [1]. The sea urchin *S. purpuratus* and the starfish *A. planci* and *P. miniata* were newly identified and added to the database. The homology percentage of all sequences was 28.28%, which was relatively low, mainly with high identity in the EF-hand conserved domain. No homologous genes have been found in vertebrates and other invertebrates. As confirmed in *H. glaberrima* [33], Aj-Orpin is a secretory protein rather than a protein targeting intracellular organelles because of the highly overlapping of the signal peptide region and the transmembrane region. Besides this, Aj-Orpin seems to be a calcium-binding protein (CaBP) containing two EF-hand. 

When studying parvalbumin by X-ray diffraction, Kretsinger proposed the EF-hand model, which has a helix-loop-helix structure [34]. The EF-hand is made up of two vertically oriented α -helices (E and F) with a calcium-binding loop of 12 amino acids between them, starting with Asp (D) and ending with Glu (E) [35,36,37,38]. The canonical EF-hand residues 1, 3, 5, 7, 9, and 12 chelate the calcium (Ca^2+^) ion, forming a pentagonal bipyramidal array of seven oxygen ligands [36]. EF-Hand CaBPs are EF-hand domain-containing proteins that may be involved in intracellular calcium-binding and calcium signal transduction, including cell proliferation and apoptosis [38]. Most known EF-hand proteins have paired 2, 4, or 6 EF-Hand structures with a strong tendency to pair and form a separate domain to allow cooperative Ca^2+^ ion binding [38,39]. Residues 111–159 in Aj-Orpin form an EF-hand pair with an odd EF-hand spanning residues 117–128 and an even EF-hand spanning residues 147–158. As many studies have shown, secreted CaBPs could promote changes in cell morphology, inhibit cell cycle, regulate extracellular matrix, and modulate cell proliferation and migration [33,40]. Osteonectin, the closest protein cluster of Orpin-like based on the phylogenetic analysis, interacts with cytokines and growth factors to play an important role in wound healing [40,41,42,43,44]. Because wound healing is a critical stage of intestinal regeneration in sea cucumber, and the cellular and molecular events that occur during tissue injury healing are similar [45], we hypothesized that Aj-Orpin functions in the wound healing stage of intestinal regeneration. Although cellular processes and protein target s for high calcium remain unclear, there is no doubt that calcium influx is necessary for cell repair [46]. In the absence of external calcium, the binding experiment of Aj-Orpin with calcium ions needs to be verified.

### 3.2. RAP May Participate in Cell Proliferation during Intestinal Regeneration

The levels of Aj-Orpin mRNA and protein expression in the intestine of *A. japonicus* at various regeneration stages were investigated. The transcription and translation levels of Aj-Orpin expression showed an inverse trend, possibly due to post-transcriptional regulation and the translation delay effect from mRNA to protein [47]. *Orpin* A and *Orpin* B expression levels in the regenerative intestine and the normal intestine were similar in *H. glaberrima*, and the expression level in the mesentery decreased during the regeneration stage [33]. In our study, the insignificant upregulation at time 0 may be due to the transient response of multiple genes at the moment of KCl stimulation. The expression of mRNA reached the first peak at 1h, perhaps because the body has just finished contracting stress at this point. Combined with protein expression levels, we speculate that Aj-Orpin responds positively during the wound healing stage and it may be related to dedifferentiation of peritoneal cell. Aj-Orpin mRNA expression peaked at 3 dpe, then gradually decreased before returning to normal at 14 dpe, which contradicted the findings in *H. glaberrima*, mainly due to differences in sampling sites and the change in the proportion of mesentery and newborn intestine. However, the protein expression level increased gradually from 3 dpe to 14 dpe, peaking on day 14. This is consistent with immunohistochemical results, which show more expression at the serosal layer and submucosa at 7 dpe compared to 3 dpe according to the staining area. The expression pattern is similar with that of WntA, an important developmental gene as well as the patterns in cell proliferation [48]. Dedifferentiation followed by mitosis, migration, and redifferentiation in the mesothelium are the most common events [4,49]. The mesothelium shows a significant increase in cell death and proliferation from 3 dpe to 14 dpe [5,31,50], indicating that Aj-Orpin may be involved in cell proliferation. The spatial expression site of Aj-Orpin is complementary to matrix metalloproteinases (MMP), like ajMMP-2 and ajMMP-16, which are mainly expressed in mucosa and submucosa [14]. This suggests that Aj-Orpin does not cooperate with MMPs to participate in the process of extracellular matrix remodeling. Consequently, the results indicate that Aj-Orpin may be connected with other developmental and growth genes to participate in cell proliferation during intestinal regeneration of the *A. japonicus*.

## 4. Materials and Methods

### 4.1. Experimental Animals and Tissue Collection

*A. japonicus* adult sea cucumbers (70–100 g) were collected off the coast of Qingdao, Shandong Province. They were immediately transported to the laboratory and acclimated for two weeks at 15–17 °C before the experiment. Evisceration was induced by injecting 3–5 mL of 0.35 M KCl into the coelom [5,31]. At each sampling stage, at least six individuals were used (control, 0 h, 30 min, 1 h, 2 h, 6 h, 12 h, 1 d, 3 d, 7 d, and 14 d post-evisceration). The sea cucumbers were temporarily kept in well-aerated in-door tanks with one individual per liter of seawater after they had completely spat out internal organs. Non-eviscerated animals were kept in the same conditions as the eviscerated animals and were fed once a day. After evisceration, we kept track of time and took residual and nascent intestinal tissues under RNase-free conditions. Before being sacrificed, all sea cucumbers were anesthetized in 6% MgCl_2_ for ~1 h [5,6]. Half of the samples were immediately frozen in liquid nitrogen and stored at −80 °C. The remaining samples were immediately fixed at each sampling point in 4% paraformaldehyde at 4 °C for 24 h. The fixative was then dehydrated in a graded series of alcohols (70%, 75%, 85%, 95%, and 100%), rinsed with xylene, and finally embedded in paraffin for tissue sectioning. Tissue sections (7 μm thickness) were adhered to slides and observed under a microscope.

### 4.2. RNA Extraction and Cloning of Full-Length Aj-Orpin Complementary DNA (cDNA)

Total RNA was extracted from the intestine using the RNeasy Mini Kit and Oligotex mRNA Kit (Qiagen, Redwood, CA, USA) according to the manufacturer’s instructions. The integrity and concentration of purified RNA were determined using 1% agarose gel electrophoresis and Nanodrop1000 (Thermo Fisher Scientific, Waltham, MA, USA).

Aj-Orpin full-length cDNA was obtained from the SMARTer^TM^ RACE cDNA Amplification Kit (Clontech, Mountain View, CA, USA), and 5′ and 3′ rapid amplification of cDNA ends polymerase chain reactions (RACE PCR). The manufacturer’s protocol was followed to obtain 5′- and 3′- RACE-Ready cDNA. Primer3 (http://www.primer3plus.com/, accessed on 17 November 2021) was used to design gene-specific primers (GSP) (GspAj-Orpin F1, GspAj-Orpin R1, GspAj-Orpin F2, and GspAj-Orpin R2) based on the sequence fragments obtained from the transcriptome library [21]. GspAj-Orpin F1, GspAj-Orpin F2, and a universal primer mix (UPM) were used to amplify the 5′ ends of Aj-Orpin (Table 1). GspAj-Orpin R1, GspAj-Orpin R2, and UPM were also used to amplify the 3′ ends of Aj-Orpin. RACE PCR amplification was performed using 5′- and 3′- RACE-Ready cDNA as templates, respectively, to obtain the 5′ and 3′ ends of Aj-Orpin. The reaction mixture and cycling parameters were followed according to the instructions (Mastercycler Pro, Eppendorf, Hamburg, Germany).

Using 1% agarose gel electrophoresis, the 5′ and 3′ PCR products were detected, and gel extraction was performed (OMEGA, USA). Following the protocol, the PCR products were inserted into the pMD19-T vector (Takara) and transformed into JM109 competent cells (Takara). The transformed competent cells were resuscitated and cultured overnight on Luria–Bertani solid medium containing 100 μg/mL ampicillin. Positive clones were selected and cultured in a liquid SOC medium for 12–16 h and then sequenced by HUADA (Wuhan, China). To obtain the full-length cDNA of Aj-Orpin, DNAStar software (DNAStar Inc., Madison, WI, USA) spliced the 5′ end sequence, known fragments, and 3′ end sequence.

### 4.3. Sequence Analysis

ORFfinder (https://www.ncbi.nlm.nih.gov/orffinder/, accessed on: 1 March 2022) was used to predict amino acid sequence and find an open reading frame (ORF) based on the full-length cDNA sequence. The Basic Local Alignment Search Tool (BLAST) at the National Center for Biotechnology Information (NCBI) was used to analyze sequence homology (https://blast.ncbi.nlm.nih.gov/Blast.cgi, accessed on: 1 March 2022). The amino acid sequence, as well as the molecular weight (MW), theoretical isoelectric point (PI), and transmembrane region, were analyzed using the ExPASy server (http://www.expasy.org/tools/, accessed on: 1 March 2022). SignalP (http://www.cbs.dtu.dk/services/SignalP/, accessed on: 1 March 2022) was used to predict signal peptides. The conserved protein domain family was predicted using NCBI’s CDD [51]. Multiple sequence alignment was performed using the Jalview software. MEGA 11.0.13 was used to create a phylogenetic tree using the Maximum Likelihood method and the Whelan And Goldman model, which involved 13 amino acid sequences [52,53].

### 4.4. Gene Expression Analysis by Real-Time Quantitative PCR (qRT-PCR)

Total RNA extraction and quality verification from the intestine at various stages of regeneration were performed using the methods mentioned above. Using Prime Script™ RT Reagent Kit with genomic DNA Eraser (Takara), RNA was reverse transcribed into single-stranded cDNA, which was used as the template for qRT-PCR. We used NADH dehydrogenase as a reference gene [23]. Table 1 lists the Aj-Orpin primers (RT-Aj-Orpin F and RT-Aj-Orpin F) and NADH primers (NADHF and NADHR). SYBR^®^ Premix ExTaq™ (Takara) was used on an Eppendorf MastercyclerHep realplex (Eppendorf, Hamburg, Germany) to assess Aj-Orpin mRNA expression levels at different regeneration stages. The reaction system was set up according to the instructions, with predenaturation at 95 °C for 5 s, followed by 40 cycles of denaturation at 95 °C for 10 s, 60 °C renaturation for the 20 s, and extension at 72 °C for 30 s. The melting curve was added as the final step to ensure that the primers were specific. The expression level of Aj-Orpin mRNA was measured using the 2^−ΔΔCt^ method.

### 4.5. Western Blotting

Total protein content was extracted from 15 mg intestine tissue, mixed with an equal volume of 2× loading buffer, and then denatured in boiling water for 5 min. We slowly added 15 μL of the mixture into the sample tank to carry out polyacrylamide gel electrophoresis until the bromophenol blue loading buffer migrated to the bottom of the gel (the 15% of the resolution gel and the 5% of the stacking gel), then transferred to PVDF membranes. Phosphate-buffered saline/0.1%Tween-20 (PBST) was used to wash the membrane three times. To block for 2 h at room temperature, 1% casein was used, then incubated with rabbit anti-Aj-Orpin antibody diluted 1:1000 with 1% casein and commercial β-actin antibody diluted 1:5000 with PBST. Horseradish peroxidase-conjugated goat anti-rabbit immunoglobulin G was used as the secondary antibody (1:2000, 1 h at room temperature). The film was exposed, developed, and fixed in a dark room. The protein expression level was normalized by comparing the density of immunoreactive bands using β-actin. Image J was used to perform the quantification analysis. The primary polyclonal antibody anti-Aj-Orpin was obtained by immunizing rabbits with synthetic specific polypeptide (CGDEKISWQEFPDMN). β-actin antibody, as a loading control, was provided by GenScript (Nanjing, China) and the procedure was completed at the GenScript biological company (Nanjing, China) for Western blotting and following immunohistochemistry.

### 4.6. Immunohistochemistry

To remove endogenous catalase, intestinal tissue sections at various stages of regeneration were dewaxed and rehydrated, then treated with 3% hydrogen peroxide for 10 min. The sections were antigen-repaired in sodium citrate buffer using a pressure cooker. They were washed in phosphate-buffered saline (PBS) and then blocked with 10% bovine serum albumin at 37 °C for 30 min after cooling to room temperature. After washing with PBS, the sections were incubated with a primary antibody against Aj-Orpin (1:100) at 4 °C overnight. The control group used PBS instead of primary antibody. The samples were then incubated with a peroxidase-conjugated secondary antibody (1:500) for 30 min at 37 °C. The color reaction was carried out using a diaminobenzidine substrate kit (Solarbio, Beijing, China). Finally, the samples were redyed with hematoxylin, dehydrated with gradient alcohol, sealed with neutral balsam, and photographed under a microscope.

### 4.7. Statistical Analysis

Using the statistical package for social sciences, a one-way analysis of variance was used for statistical analysis. GraphPad Prism 9.0.0.121 was used to create histograms. All data are presented as the mean ± standard error (*n* = 3) with a difference significance level of *p* < 0.05.

## 5. Conclusions

In this study, we cloned and characterized the full-length cDNA of Aj-Orpin, which was 1136 bp long and contained a 22 bp 5′-UTR, a 619 bp 3′-UTR, and a 495 bp ORF encoding a 164 amino acid protein. Aj-Orpin was discovered to be a two-copy gene found only in echinoderms and *Saccoglossus kowalevskii*. Because of the EF-hand domain, Aj-Orpin appears to be linked to calcium-binding and plays a specific role in intestinal regeneration. According to the spatiotemporal expression, Aj-Orpin has opposite expression patterns at the mRNA and protein levels. This could be caused by post-transcriptional regulation or the interaction of double-copy genes. Protein expression levels were significantly higher during blastema formation (3–7 dpe) and lumen formation (7–14 dpe). Aj-Orpin was highly expressed in the serous layer and submucosa, suggesting that it may work in tandem with cell proliferation. Additional research is needed to fully understand Aj-Orpin’s role in intestinal regeneration.

## Figures and Tables

**Table 1:** Sequences of RACE PCR and real-time PCR primers.

Primer	Sequence
GspAj-Orpin F1	5′- GACCCACCCCAACCCGATTC -3′
GspAj-Orpin R1	5′- GCTTTGCCGCCCTCTTTCGT -3′
GspAj-Orpin F2	5′- GGTGGATGGTTGAAGAGAAGTTGGAAG -3′
GspAj-Orpin R2	5′- CCCCACTTCGTCACGTTGATATTTACC -3′
UPM	Long5′-CTAATACGACTCACTATAGGGCAAGCAGTGGTATCAACGCAGAGT-3′ Short5′-CTAATACGACTCACTATAGGGC-3′
RT-Aj-Orpin F	5′-AGTGGTGCTGTTGGTGTTAAGGTC-3′
RT-Aj-Orpin R	5′-CCGCCCTCTTTCGTCCATTCAC-3′
NADHF	5′-GTCCTACGACCCAATCTGGA-3′
NADHR	5′-ATGAGCCTTGGTTACGTTGG-3′

## Data Availability

Not applicable.
